# Impact of insulin resistance on subclinical left ventricular dysfunction in normal weight and overweight/obese japanese subjects in a general community

**DOI:** 10.1186/s12933-020-01201-6

**Published:** 2021-01-21

**Authors:** Kazutoshi Hirose, Koki Nakanishi, Masao Daimon, Naoko Sawada, Yuriko Yoshida, Kentaro Iwama, Yuko Yamamoto, Jumpei Ishiwata, Megumi Hirokawa, Katsuhiro Koyama, Tomoko Nakao, Hiroyuki Morita, Marco R. Di Tullio, Shunichi Homma, Issei Komuro

**Affiliations:** 1 Department of Cardiovascular Medicine, The University of Tokyo, Tokyo, Japan; 2 Department of Clinical Laboratory, The University of Tokyo, Tokyo, Japan; 3 Department of Medicine, Columbia University, New York, NY USA

**Keywords:** Insulin resistance, Left ventricular global longitudinal strain, Speckle‐tracking echocardiography

## Abstract

**Background:**

Insulin resistance carries increased risk of heart failure, although the pathophysiological mechanisms remain unclear. LV global longitudinal strain (LVGLS) assessed by speckle-tracking echocardiography has emerged as an important tool to detect early LV systolic abnormalities. This study aimed to investigate the association between insulin resistance and subclinical left ventricular (LV) dysfunction in a sample of the general population without overt cardiac disease.

**Methods:**

We investigated 539 participants who voluntarily underwent extensive cardiovascular health check including laboratory test and speckle-tracking echocardiography. Glycemic profiles were categorized into 3 groups according to homeostatic model assessment of insulin resistance (HOMA-IR): absence of insulin resistance (HOMA-IR < 1.5), presence of insulin resistance (HOMA-IR ≥ 1.5) and diabetes mellitus (DM). Multivariable logistic regression models were conducted to evaluate the association between abnormal glucose metabolism and impaired LVGLS (> − 16.65%).

**Results:**

Forty-five (8.3%) participants had DM and 66 (12.2%) had abnormal HOMA-IR. LV mass index and E/e′ ratio did not differ between participants with and without abnormal HOMA-IR, whereas abnormal HOMA-IR group had significantly decreased LVGLS (− 17.6 ± 2.6% vs. − 19.7 ± 3.1%, p < 0.05). The prevalence of impaired LVGLS was higher in abnormal HOMA-IR group compared with normal HOMA-IR group (42.4% vs. 14.0%) and similar to that of DM (48.9%). In multivariable analyses, glycemic abnormalities were significantly associated with impaired LVGLS, independent of traditional cardiovascular risk factors and pertinent laboratory and echocardiographic parameters [adjusted odds ratio (OR) 2.38, p = 0.007 for abnormal HOMA-IR; adjusted OR 3.02, p = 0.003 for DM]. The independent association persisted even after adjustment for waist circumference as a marker of abdominal adiposity. Sub-group analyses stratified by body mass index showed significant association between abnormal HOMA-IR and impaired LVGLS in normal weight individuals (adjusted OR 4.59, p = 0.001), but not in overweight/obese individuals (adjusted OR 1.62, p = 0.300).

**Conclusions:**

In the general population without overt cardiac disease, insulin resistance carries independent risk for subclinical LV dysfunction, especially in normal weight individuals.

## Background

Type 2 diabetes mellitus (DM) affects an increasing number of people worldwide. In the United States, 26 million adults suffer from DM, with new diagnoses occurring in approximately 1.5 million per year [1]. DM causes atherosclerotic cardiovascular disease and subsequent left ventricular (LV) dysfunction. Furthermore, DM per se may cause myocardial impairment known as “diabetic cardiomyopathy”, leading to two to fourfold increased risk of heart failure (HF) compared with nondiabetic individuals [2–7]. Given the unfavorable outcomes and enormous economic burden, early identification of individuals at higher risk for HF and timely therapeutic intervention is of crucial importance. Two-dimensional speckle-tracking echocardiography is a reliable method for the objective quantification of early LV dysfunction with excellent feasibility and reproducibility [8]. Impairment of LV global longitudinal strain (LVGLS) precedes LV ejection fraction decrease, and was an independent and more sensitive marker of incident HF in various clinical settings including DM [9–12].

Recent population-based cohort studies identified even mild insulin resistance as an independent and significant risk factor for incident HF [13–16]. In the Atherosclerosis Risk in Communities (ARIC) study, homeostatic model assessment of insulin resistance (HOMA-IR) ≥ 1.5 carries a significant risk of HF development independent of traditional risk factors [16]. Despite the impact of abnormal glucose metabolism on HF and the possibility of measuring LV strain, the association between insulin resistance and subclinical LV dysfunction is not fully studied. Understanding the association between insulin resistance and subclinical LV dysfunction might provide insight into the pathogenesis of diabetic cardiomyopathy and may help inform the possible preventive strategies for HF caused by abnormal glucose metabolism. Therefore, the present study aimed to determine whether insulin resistance assessed by HOMA-IR carries an independent risk for impaired LVGLS in the general population without prevalent cardiovascular disease.

## Methods

### Study participants

We included 572 consecutive asymptomatic participants who voluntarily underwent extensive cardiovascular health check, including laboratory testing and 2-dimensional echocardiography, between June 2018 and May 2019. Our clinic provides an extensive health check for the promotion of health and prevention of cardiovascular disease. Subjects with atrial fibrillation or flutter (n = 7), history of coronary artery disease (n = 17), decreased LV systolic function (LV ejection fraction < 50%) or moderate or severe aortic/mitral valvular disease (n = 6) or inadequate image quality of the echocardiographic examination (n = 3) were excluded. Thus, the final study group comprised 539 subjects without overt cardiac disease. All participants provided informed consent that allowed all de-identified data including laboratory test and echocardiographic examination to be used for research purpose at the time of health check-up, and included an opt-out option for analyses afterwards. The Institutional Review Boards of the University of Tokyo approved the study.

### Risk factor assessment

All participants underwent a medical evaluation of clinical history and physical examination with anthropometrics and blood pressure measurement. Hypertension was defined as systolic blood pressure ≥ 140 mm Hg or diastolic blood pressure ≥ 90 mm Hg, or receiving antihypertensive medications. Diabetes mellitus (DM) was defined by a fasting glucose ≥ 126 mg/dl or current use of insulin or hypoglycemic agents. Hyperlipidemia was defined as total serum cholesterol > 240 mg/dl, or the use of lipid-lowering drugs. Body mass index (BMI) was calculated using height and weight (kg/m^2^) and the waist circumference (WC) was measured at the level of the umbilicus.

### Laboratory testing and classification of glucose metabolism

Venous blood samples were drawn in the fasting condition on the same day as the echocardiographic examination. Fasting serum glucose, insulin, total cholesterol, low-density lipoprotein cholesterol, high-density lipoprotein cholesterol and C-reactive protein were measured in all participants. HOMA-IR was calculated from the following validated formula; HOMA-IR = fasting insulin (µU/ml) × fasting blood glucose (mg/dl)/405 [17]. Participants without DM were classified into 2 groups according to HOMA-IR; participants with insulin resistance as HOMA-IR ≥ 1.5 and those without insulin resistance as HOMA-IR < 1.5, which is based on the cut-off value carrying increased risk of incident heart failure from ARIC Study as mentioned above [16]. Overall, glycemic profiles were categorized into 3 groups: (1) normal HOMA-IR group (no DM and HOMA-IR < 1.5); (2) abnormal HOMA-IR group (no DM and HOMA-IR ≥ 1.5); and (3) DM group.

### Echocardiography

#### Standard echocardiography

All participants underwent standard two-dimensional transthoracic echocardiographic examination using a commercially available system (Toshiba Aplio, Toshiba Medical System Corp, Tochigi, Japan) by trained and registered cardiac sonographers who were blinded to other clinical information. The dimensions of cardiac chambers were measured in the standard manner [18]. LV mass was calculated with a validated formula:

1 $${\text{LV mass}} = 0.8(1.04[({\text{IVST}}+{\text{LVEDD}}+{\text{PWT}})^3-{\text{LVEDD}}^3])+0.6$$

where IVST is the end-diastolic interventricular septal thickness, LVEDD: LV end-diastolic diameter, and PWT is the end-diastolic posterior wall thickness [19]. Left atrial volume was evaluated by the biplane Simpson’s rule. LV mass and left atrial volume were then indexed to body surface area. Transmitral blood flow signals were used to measure peak early (E) and late (A) diastolic velocity. Using tissue Doppler imaging, early peak diastolic velocity (e′) of the septal and lateral mitral annulus was measured and averaged. E/e′ ratio was then calculated.

#### Speckle‐tracking echocardiography

Speckle-tracking analysis was performed offline using vendor-independent and commercially available software (2D Cardiac Performance Analysis; Tomtec Imaging System, Germany). Semi-automated border detection was performed, and LV border was tracked throughout the cardiac cycle. Manual correction was performed in case of inadequate endocardial detection. LV global longitudinal strain (LVGLS) was obtained by averaging negative peak of segmental strain values from all 3 apical views, including the 4-chamber, 2-chamber, and long-axis views [8]. Abnormal LVGLS was defined as a GLS > − 16.65%, which was the 90th percentile of the strain value distribution in the study participants without any conditions associated with LV remodeling including hypertension, diabetes mellitus, coronary artery disease, arrhythmias, significant valvular disease or BMI > 25 kg/m^2^. This cutoff value was consistent with previous studies exploring normal LVGLS [20, 21]. According to the definition of strain, negative strain denotes shortening for LV which indicates that increasing absolute values represent a better ventricular function. Excellent correlations were observed in the inter- and intra-observer variabilities of LVGLS in 15 randomly selected participants (r = 0.93 and r = 0.94, respectively). In the Bland-Altman analysis, the inter- and intra-observer variabilities were − 0.6 ± 1.3% and 0.2 ± 1.4% (mean ± 1.96 standard deviation, respectively). All echocardiographic analyses were performed by KH, KN and NS who were blinded to the participants’ metabolic profiles.

### Statistical analysis

Continuous variables were presented as mean ± standard deviation or median (interquartile range) and compared using the Analysis of variance with Tukey–Kramer post hoc analysis or a Kruskal–Wallis test with the post-test Dunn correction as appropriate. Categorical variables were described as numbers and proportions, and compared using the chi-square test. Univariable correlation between HOMA-IR/insulin level and LVGLS was evaluated by Pearson’s correlation coefficients (r). Univariable and multivariable logistic regression models were constructed to investigate the association between abnormal glucose metabolism and impaired LVGLS (> − 16.65%) with adjustment for the following covariates: Model 1: adjustment for age and sex; Model 2: adjustment for age, sex, hypertension, hyperlipidemia, smoking status and BMI (BMI-adjusted model) or WC (WC-adjusted model); Model 3: model 2 plus echocardiographic parameters including LV mass index and E/e′; Model 4: model 3 plus biomarkers (i.e. estimated glomerular filtration rate and serum C-reactive protein). Covariates were selected on the basis of possible clinical relevance and known association with LV dysfunction. Adjusted odds ratios (ORs) with their 95% confidence interval (CI) were calculated in the entire study group and in BMI subgroups: normal weight (BMI < 25 kg/m^2^) and overweight or obese group (BMI ≥ 25 kg/m^2^). The incremental value of HOMA-IR over baseline characteristics was also assessed by comparison of Chi-square values. A value of p < 0.05 was considered significant. All analyses were performed with the JMP 14 statistical software (SAS Institute, Inc, Cary, NC, USA).

## Results

### Baseline characteristics

**Fig. 1:**
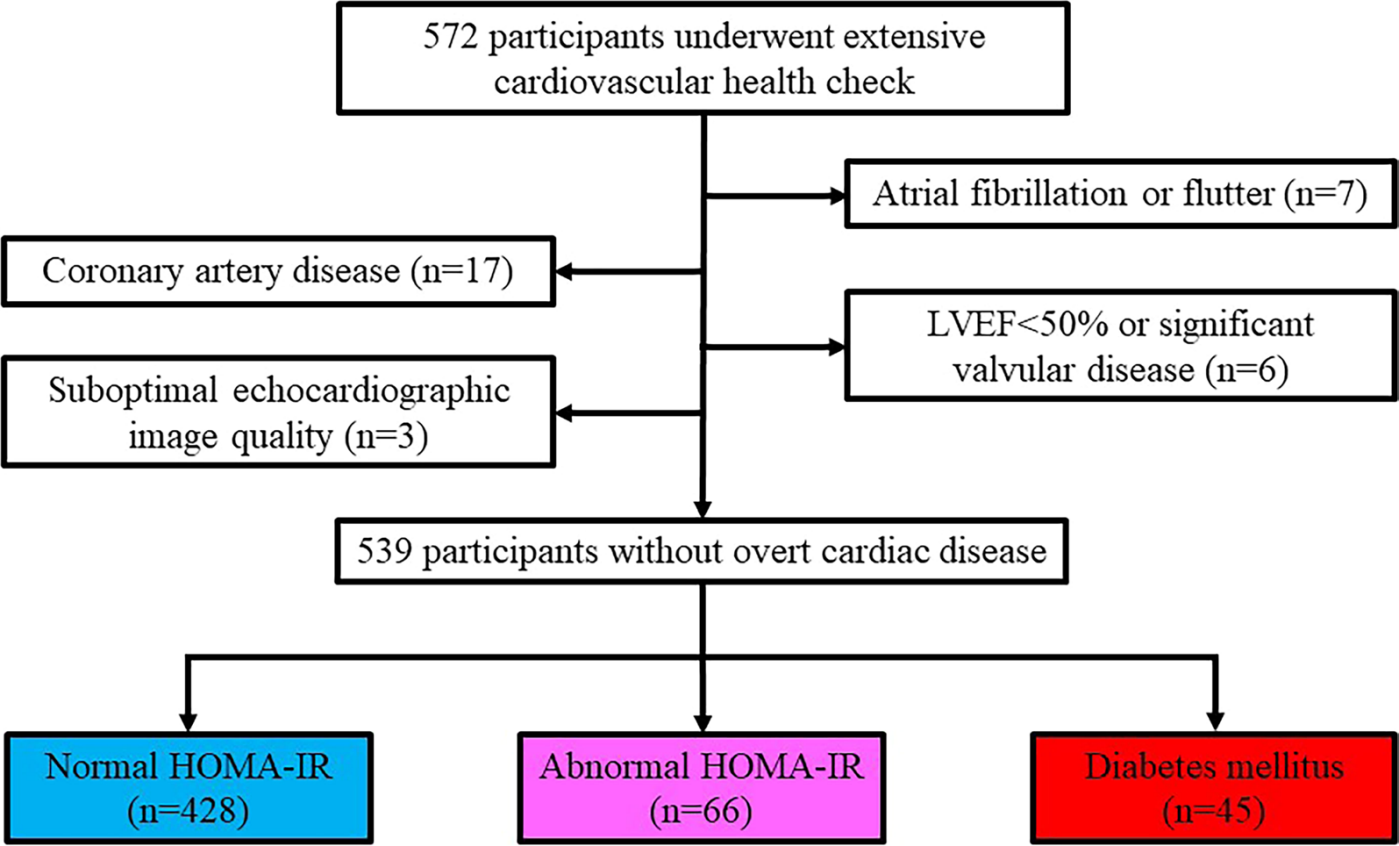
Flowchart of study population. *HOMA-IR* homeostatic model assessment of insulin resistance, *LVEF* left ventricular ejection fraction

Clinical characteristics and echocardiographic parameters of the study population are shown in Table 1. Mean age was 57 ± 10 years, and 370 (68.6%) were men. Mean LV ejection fraction was 62.5 ± 5.6% and LVGLS was −19.2 ± 3.1%. Forty-five (8.3%) participants had diabetes mellitus and 66 (12.2%) had abnormal HOMA-IR (Fig. 1). All major cardiovascular risk factors showed significant correlations with glycemic abnormalities (also Table 1).

**Table 1:** Characteristics of the study population stratified by glucose metabolism

	Normal HOMA-IR (n = 428)	Abnormal HOMA-IR (n = 66)	DM (n = 45)	p value
Age, years	56 ± 10	57 ± 10	62 ± 9*^,†^	0.002
Men, n (%)	269 (62.9)	60 (90.9)	41 (91.1)	< 0.001
Hypertension, n (%)	101 (23.6)	29 (43.9)	25 (55.6)	< 0.001
Hyperlipidemia, n (%)	131 (30.6)	30 (45.5)	33 (73.3)	< 0.001
Current smoking, n (%)	88 (20.6)	18 (27.3)	13 (28.9)	0.244
Body mass index, kg/m^2^	23.0 ± 2.8	26.4 ± 2.9*	24.9 ± 3.7*^,†^	< 0.001
Overweight/obesity, n (%)	100 (23.4)	40 (60.6)	17 (37.8)	< 0.001
Waist circumference, cm	83.9 ± 8.0	94.1 ± 8.0*	91.2 ± 8.9*	< 0.001
Systolic blood pressure, mm Hg	118 ± 15	124 ± 12*	128 ± 16*	< 0.001
Diastolic blood pressure, mm Hg	76 ± 10	80 ± 10*	78 ± 9	< 0.001
Medications				
Anti-hypertensive drugs, n (%)	66 (15.4)	20 (30.3)	22 (48.9)	< 0.001
Lipid-lowering drugs, n (%)	76 (17.8)	22 (33.3)	29 (64.4)	< 0.001
Oral anti-diabetic drugs, n (%)	0 (0)	0 (0)	37 (82.2)	N/A
Laboratory parameters				
Fasting glucose, mg/dl	92 ± 8	101 ± 9*	118 ± 20*^,†^	< 0.001
Fasting Insulin, μU/ml	3.02 ± 1.45	8.73 ± 2.83*	7.84 ± 16.42*	< 0.001
HOMA-IR	0.70 ± 0.35	2.17 ± 0.69*	2.16 ± 3.55*	< 0.001
HbA1c, %	5.6 ± 0.3	5.8 ± 0.4*	6.9 ± 0.7*^,†^	< 0.001
Total cholesterol, mg/dl	205 ± 34	207 ± 40	184 ± 37*^,†^	< 0.001
LDL cholesterol, mg/dl	121 ± 31	124 ± 31	106 ± 34*^,†^	0.006
HDL cholesterol, mg/dl	68 ± 17	54 ± 13*	57 ± 14*	< 0.001
eGFR, ml/min/1.73m^2^	77 ± 14	75 ± 16	75 ± 18	0.255
C-reactive protein, mg/dl	0.04 (0.02–0.08)	0.07 (0.05–0.11)*	0.07 (0.03–0.14)*	< 0.001
Echocardiographic parameters				
LV end-diastolic diameter, mm	44.1 ± 4.1	45.8 ± 4.7*	45.7 ± 3.9*	0.001
LV end-systolic diameter, mm	28.1 ± 3.2	29.3 ± 3.8*	29.3 ± 3.2	0.004
LV ejection fraction, %	63.0 ± 5.5	60.4 ± 5.3*	60.3 ± 5.3*	< 0.001
LV mass index, g/m^2^	64.5 ± 15.2	66.4 ± 15.1	72.8 ± 17.6*	0.002
E wave, cm/s	66.1 ± 13.6	61.9 ± 9.9*	58.3 ± 11.4*	< 0.001
A wave, cm/s	57.7 ± 14.7	59.3 ± 14.2	62.1 ± 14.5	0.130
E/A ratio	1.21 ± 0.39	1.10 ± 0.30*	0.97 ± 0.25*	< 0.001
e′, cm/s	8.2 ± 2.0	7.4 ± 1.6*	6.5 ± 1.4*^,†^	< 0.001
E/e′ ratio	8.4 ± 2.1	8.7 ± 2.0	9.3 ± 2.1*	0.017
LA volume index, ml/m^2^	22.0 ± 5.7	23.0 ± 6.1	21.9 ± 5.0	0.359
LVGLS, %	− 19.7 ± 3.1	− 17.6 ± 2.6*	− 16.8 ± 2.4*	< 0.001
Impaired LVGLS, n (%)	60 (14.0)	28 (42.4)	22 (48.9)	< 0.001

Values are mean ±  standard deviation, n (percentage), or median (25th–75th percentile)

*A* late diastolic transmitral flow velocity, *DM* diabetes mellitus, *E* early diastolic transmitral flow velocity, *e′* early diastolic mitral annular velocity, *eGFR* estimated glomerular filtration rate, *GLS* global longitudinal strain, *HDL* high density lipoprotein, *HOMA-IR* homeostatic model assessment of insulin resistance, *LA* left atrium, *LDL* low density lipoprotein, *LV* left ventricle

* p<0.05 compared with normal HOMA-IR group

^†^p<0.05 compared with abnormal HOMA-IR group

### Abnormal glucose metabolism and LVGLS

**Fig. 2:**
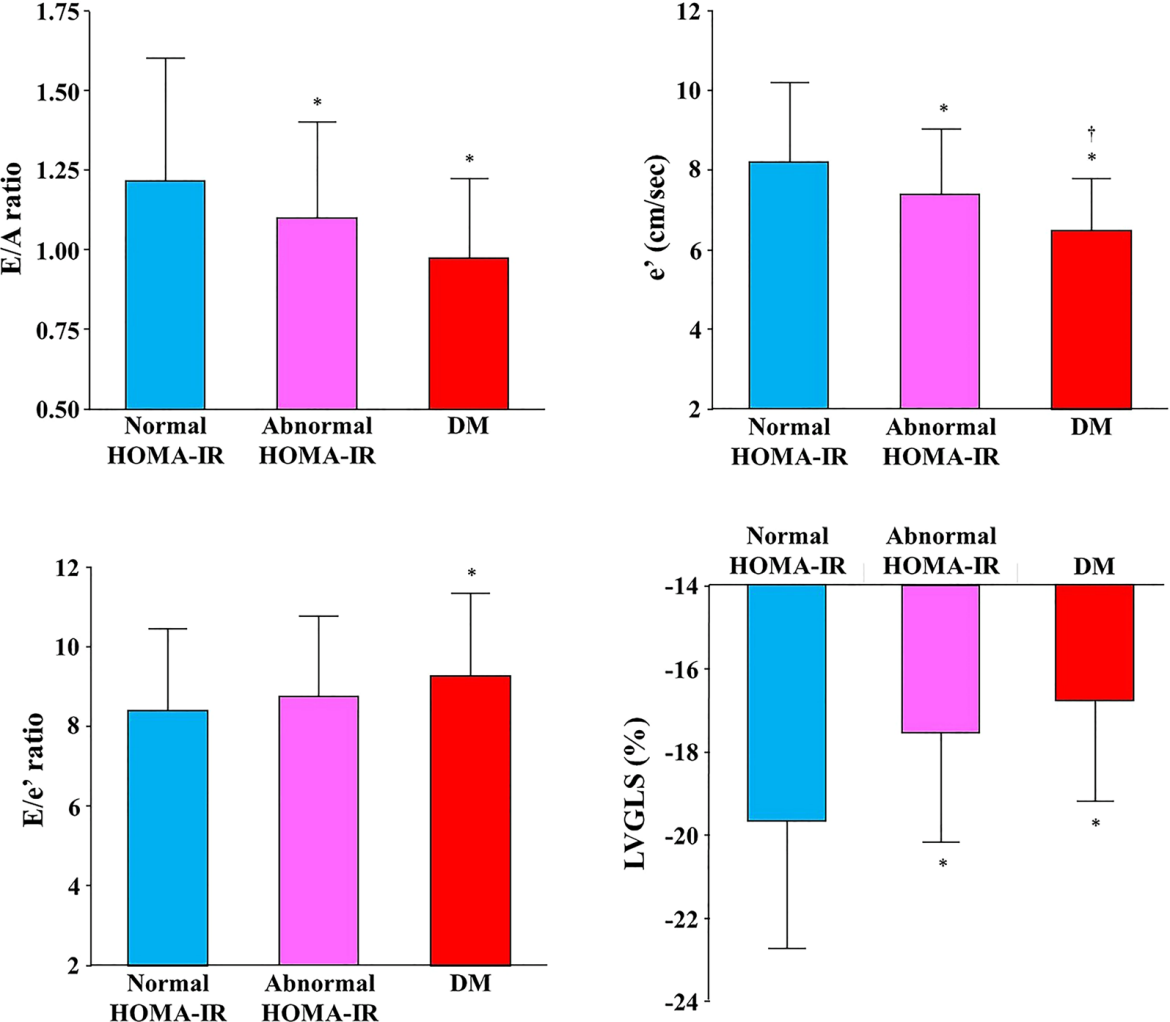
Relationship between glycemic profiles and echocardiographic parameters. *A* late diastolic transmitral flow velocity, *DM* diabetes mellitus, *E* early diastolic transmitral flow velocity, *e*’ early diastolic mitral annular velocity, *HOMA-IR* homeostatic model assessment of insulin resistance, *LVGLS* left ventricular global longitudinal strain. *p < 0.05 compared with normal HOMA-IR group. ^†^p < 0.05 compared with abnormal HOMA-IR group

**Fig. 3:**
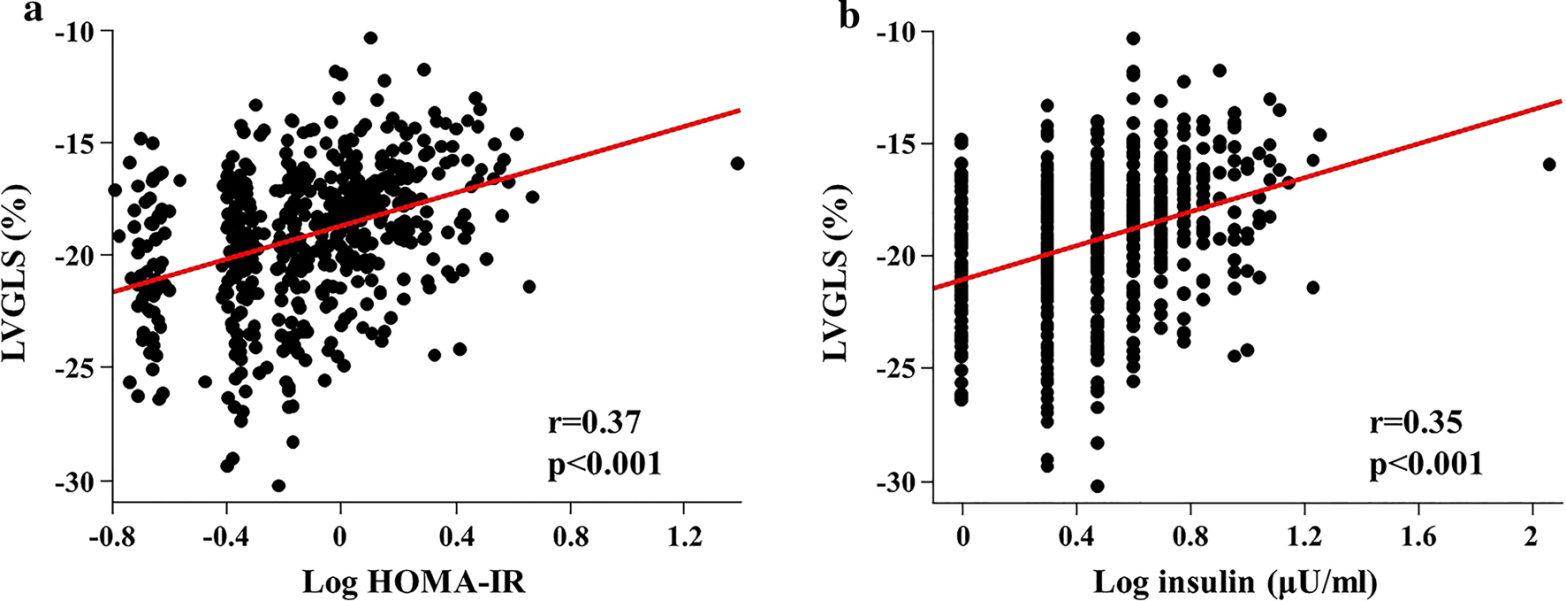
Scatter plots illustrating the association of LVGLS with HOMA-IR (**a**) and insulin level (**b**). *HOMA-IR* homeostatic model assessment of insulin resistance, *LVGLS* left ventricular global longitudinal strain

**Fig. 4:**
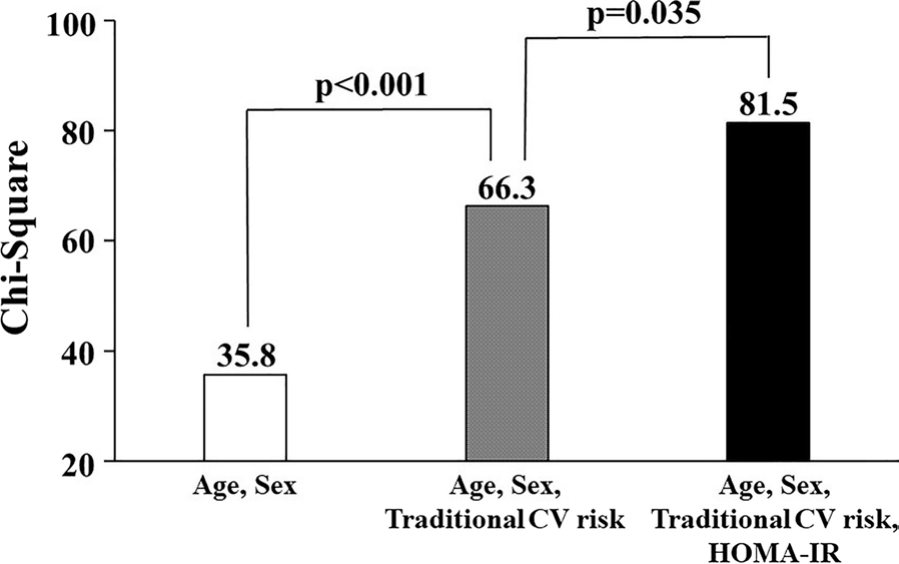
Incremental value of insulin resistance for the identification of impaired LVGLS. Traditional CV risk includes hypertension, hyperlipidemia. diabetes mellitus, current smoking and obesity. *CV* cardiovascular, *HOMA-IR* homeostatic model assessment of insulin resistance, *LVGLS* left ventricular global longitudinal strain

Significantly larger LV mass index and elevated E/e′ ratio were observed in individuals with DM compared with those with normal glucose metabolism, whereas there was no significant difference between those with and without abnormal HOMA-IR (Table 1). On the other hand, participants with abnormal HOMA-IR had significantly decreased LVGLS compared with normal HOMA-IR group ( −17.6 ± 2.6% vs. − 19.7 ± 3.1%, p < 0.05; Fig. 2). There was a significant difference in e′ between abnormal HOMA-IR group and DM group, whereas E/A ratio, E/e′ ratio and LVGLS did not differ between the 2 groups. The prevalence of impaired LVGLS (> − 16.65%) was greatest in DM group (48.9%), followed by abnormal HOMA-IR group (42.4%) and normal HOMA-IR group (14.0%, overall p < 0.001; Table 1). Higher HOMA-IR value and serum insulin level were significantly related to decreased LVGLS (r = 0.37 and r = 0.35, both p < 0.001; Fig. 3a, b). Table 2 demonstrates the association between glycemic abnormality and impaired GLS. Both abnormal HOMA-IR and DM were associated with impaired LVGLS in the age- and sex-adjusted model (Model 1). In the multivariable model adjusted for age, sex, hypertension, hyperlipidemia, smoking status and BMI, this association persisted (Model 2). With further adjustment for echocardiographic parameters including LV mass index and E/e′, abnormal HOMA-IR and DM remained significantly associated with LVGLS (Model 3). Even after controlling for biomarkers of estimated glomerular filtration rate and C-reactive protein (Model 4), glycemic abnormality was related to subclinical LV dysfunction (adjusted OR: 2.38, p = 0.007 for abnormal HOMA-IR and adjusted OR 3.02, p = 0.003 for DM). When WC was entered as covariate instead of BMI, similar results were obtained: abnormal HOMA-IR carried independent risk for abnormal LVGLS in the full-adjusted model (adjusted OR 2.23, p = 0.013; also Table 2). HOMA-IR was a significant predictor for impaired LVGLS and produced a significant increase in model Chi-square over baseline characteristics (p = 0.035; Fig. 4).

**Table 2:** Association between glycemic abnormality and impaired LVGLS (> − 16.65%)

	Abnormal HOMA-IR		DM	
	Odds ratio (95% CI)	p value	Odds ratio (95% CI)	p value
Univariable	4.52 (2.58–7.91)	< 0.001	5.87 (3.08–11.2)	< 0.001
Model 1	3.49 (1.97–6.20)	< 0.001	4.05 (2.07–7.92)	< 0.001
BMI adjusted model				
Model 2	2.34 (1.26–4.34)	0.007	2.97 (1.45–6.09)	0.003
Model 3	2.37 (1.27–4.42)	0.006	2.95 (1.44–6.06)	0.003
Model 4	2.38 (1.27–4.44)	0.007	3.02 (1.46–6.24)	0.003
WC adjusted model				
Model 2	2.21 (1.17–4.15)	0.014	2.75 (1.33–5.68)	0.006
Model 3	2.24 (1.19–4.23)	0.013	2.73 (1.32–5.63)	0.007
Model 4	2.23 (1.18–4.22)	0.013	2.80 (1.35–5.81)	0.006

Reference; normal HOMA-IR

Model 1: adjusted for age and sex

Model 2: adjusted for Model 1 plus hypertension, hyperlipidemia, smoking status and BMI (BMI adjusted model) or WC (WC-adjusted model)

Model 3: adjusted for Model 2 plus echocardiographic parameters including LV mass index and E/e′

Model 4: adjusted for Model 3 plus serum CRP and eGFR

*BMI* body mass index, *CI* confidence interval, *CRP* C-reactive protein, *DM* diabetes mellitus, *E*  early diastolic transmitral flow velocity, *e′* early diastolic mitral annular velocity, *eGFR * estimated glomerular filtration rate, *GLS* global longitudinal strain, *HOMA-IR * homeostatic model assessment of insulin resistance, *LV* left ventricle, *WC* waist circumference

### Impact of BMI on glucose metabolism and LVGLS

Finally, we examined the impact of abnormal glucose metabolism on subclinical LV dysfunction in BMI subgroups. Participants were classified into 2 groups; normal weight (BMI < 25 kg/m^2^) and overweight/obesity (BMI ≥ 25 kg/m^2^). In the multivariable analyses, abnormal HOMA-IR was independently associated with impaired LVGLS in normal weight group (adjusted OR 4.59, p = 0.001), but not in overweight/obese group (adjusted OR 1.62, p = 0.300; Table 3). On the other hand, DM carries independent risk of impaired LVGLS in both normal weight and overweight/obese individuals.

**Table 3:** Association between glycemic abnormality and impaired LVGLS (> − 16.65%) stratified by BMI category

	BMI < 25 kg/m^2^				BMI ≥ 25 kg/m^2^			
	Abnormal HOMA-IR		DM		Abnormal HOMA-IR		DM	
	Odds ratio (95% CI)	p value	Odds ratio (95% CI)	p value	Odds ratio (95% CI)	p value	Odds ratio (95% CI)	p value
Univariable	7.66 (3.27–17.94)	< 0.001	5.78 (2.50–13.39)	< 0.001	1.80 (0.83–3.90)	0.134	4.96 (1.67–14.72)	0.004
Model 1	6.01 (2.52–14.33)	< 0.001	3.93 (1.63–9.48)	0.002	1.69 (0.77–3.71)	0.194	4.27 (1.40–13.01)	0.011
Model 2	4.66 (1.90–11.39)	< 0.001	3.00 (1.18–7.61)	0.021	1.43 (0.60–3.41)	0.415	3.40 (0.98–11.76)	0.054
Model 3	4.65 (1.89–11.44)	< 0.001	3.02 (1.16–7.82)	0.023	1.54 (0.64–3.73)	0.337	4.08 (1.14–14.57)	0.031
Model 4	4.59 (1.84–11.44)	0.001	2.93 (1.11–7.75)	0.030	1.62 (0.65–4.05)	0.300	5.02 (1.33–18.92)	0.017

Reference; normal HOMA-IR

Model 1: adjusted for age and sex

Model 2: adjusted for Model 1 plus BMI, hypertension, hyperlipidemia and smoking status

Model 3: adjusted for Model 2 plus echocardiographic parameters including LV mass index and E/e′

Model 4: adjusted for Model 3 plus serum CRP and eGFR

*BMI * body mass index, *CI* confidence interval, *CRP* C-reactive protein, *DM* diabetes mellitus, *E* early diastolic transmitral flow velocity, *e′* early diastolic mitral annular velocity, *eGFR* estimated glomerular filtration rate, *GLS* global longitudinal strain, *HOMA-IR* homeostatic model assessment of insulin resistance, *LV* left ventricle

## Discussion

The present study is the first to report that: (i) approximately 40% non-diabetic individuals with abnormal HOMA-IR had impaired LVGLS in a sample of the general population free of overt cardiac disease, (ii) abnormal HOMA-IR was significantly associated with impaired LVGLS independent of traditional cardiovascular risk factors as well as pertinent laboratory and echocardiographic parameters, (iii) the independent association remained significant even after adjustment for WC as a marker of abdominal adiposity, and (iv) abnormal HOMA-IR may carry a different risk for impaired LVGLS depending on BMI.

DM causes LV dysfunction even without overt coronary artery disease (i.e. diabetic cardiomyopathy), leading to higher incidence of HF compared with non-diabetic individuals [2–7]. Moreover, recent population-based studies showed significantly elevated risk of HF in non-diabetic individuals with insulin resistance [13–16]. ARIC study showed that HOMA-IR ≥ 1.5 carrying significant risk for incident HF independent of traditional risk factors [16]. The Third National Health and Nutrition Examination Survey (NHANES III) also reported HOMA-IR > 1.4 accounting for a 2.6- to 3.7-fold increased risk of cardiovascular death including HF [14]. Echocardiographic studies demonstrated the association between insulin resistance and unfavorable LV morphology and functional remodeling [22, 23]. Elevated HOMA-IR was associated with LV hypertrophy in 2623 Framingham Heart Study participants [22]. The Echocardiographic Study of Latinos (ECHO-SOL) study also demonstrated that higher HOMA-IR was correlated with concentric remodeling and elevated E/e′ ratio in 1818 participants [23]. However, LV hypertrophy and diastolic dysfunction were attributed to extracellular matrix remodeling with collagen deposition and fibrosis [24], therefore suggesting the presence of partially irreversible conditions with limited effect of therapeutic intervention [25]. LVGLS assessed by two-dimensional speckle-tracking echocardiography is an early and sensitive marker of LV dysfunction, allowing more accurate prediction for incident HF in various clinical settings compared with conventional parameters [9–12]. Our observations expand the results of previous epidemiological studies by showing the association of insulin resistance with unfavorable LV remodeling and subsequent HF risk to preclinical settings. Considering the huge global burden of abnormal glucose metabolism and HF, insulin resistance may be a crucial therapeutic target for HF prevention.

Several possible mechanisms might account for the independent association between insulin resistance and subclinical LV dysfunction. First, chronic inflammation was observed in individuals with higher HOMA-IR [14], which may deteriorate LV systolic function [26]. Indeed, a recent study demonstrated a significant association between soluble receptor for advanced glycation end products (sRAGE), as a marker related to inflammation, and LV remodeling [27]. Second, activation of the renin angiotensin system accompanied by elevated HOMA-IR [28] might cause reduced LVGLS [29]. Finally, impaired coronary flow reserve was reported in individuals with abnormal HOMA-IR [30], which might be involved in the association between insulin resistance and impaired LVGLS [31, 32]. Recent studies have reported on the association between insulin resistance, dysglycemia and subclinical LV dysfunction in some clinical settings [33–37]. Ho et al. showed an inverse relationship between HOMA-IR and LVGLS in the Framingham Heart Study [33]. Kishi et al. demonstrated that higher HOMA-IR (4.7 ± 2.2) was associated with deterioration of LV strain in young adults [34]. Our findings are in line with these studies while adding that even individuals with mild insulin resistance (HOMA-IR ≥ 1.5) have subclinical LV dysfunction; in fact, the frequency of impaired LVGLS in them was similar to that in DM patients (42.4% vs. 48.9%). Furthermore, we observed that the association between HOMA-IR and LVGLS was independent of traditional cardiovascular risk factors, pertinent laboratory parameters, and LV geometry and diastolic parameters, which represents novel information.

Although abdominal adiposity is strongly correlated with insulin resistance as well as LV function [38], the association between abnormal HOMA-IR and impaired LVGLS was independent of WC in the present study. This is partially explained by the direct effect of insulin resistance on myocardial mechano-energetic efficiency, namely the ratio between myocardial external work and oxygen consumption [39]. Insulin resistance causes reduction in myocardial glucose transporter expression and shift toward fatty acid metabolism which results in higher oxygen consumption, increased oxidative stress, impaired cardiomyocyte calcium handling, and subsequent contractile dysfunction [3, 40]. In addition, excessive free fatty acid uptake also leads to myocardial triglyceride accumulation and production of lipotoxic intermediates promoting cardiomyocyte apoptosis [3, 6].

We also demonstrated that abnormal HOMA-IR carries independent risk for impaired LVGLS in normal weight individuals but not in those overweight or obese. One possible mechanism is the different impact of insulin resistance on cardiovascular profiles according to BMI. Previous studies showed that lean subjects with insulin resistance have an unfavorable inflammatory profile with elevated tumor necrosis factor-α and interleukin-6, while obese subjects exhibited a comparable inflammatory status regardless of insulin resistance [41, 42]. In addition, obese individuals often have hypertension and sleep disorders such as sleep apnea, which can deteriorate LV function and may attenuate the association between insulin resistance and subclinical LV dysfunction [31, 43, 44]. Indeed, abnormal HOMA-IR was reported to be an independent predictor of cardiovascular mortality in normal weight individuals but not in overweight/obese individuals in the community-based cohort study [45].

### Clinical implication

The novelty of the present study is that abnormal HOMA-IR carried a significant risk for subclinical LV dysfunction independent of traditional cardiovascular risk factors, LV mass and diastolic parameters, as well as WC as a marker of abdominal obesity. In addition, the independent association was observed in normal weight subjects but not in overweight/obese subjects. Our findings provide valuable information to clarify the underlying mechanism linking insulin resistance and incident HF. Furthermore, they emphasize the importance of an early detection of LV dysfunction for possible preventive strategies in individuals with abnormal HOMA-IR, particularly in normal weight subjects. Future studies are warranted to elucidate whether therapeutic interventions such as exercise and dietary counselling may have beneficial effects on subclinical LV dysfunction and possibly prevent HF development.

### Study limitation

Several limitations should be noted. First, the numbers of subjects with abnormal HOMA-IR and DM are significantly smaller than those with normal HOMA-IR and we were not able to draw causal inferences between insulin resistance and subclinical LV dysfunction because of the observational and cross-sectional nature of the study. In addition, the number of overweight/obese subjects is relatively small in the present study; Asians tend to have smaller BMI compared with Westerners, which might not allow generalization of our results to cohorts with different demographic composition. Second, the definition of diabetes mellitus is based on the fasting glucose level measured once at the time of echocardiography or the current use of hypoglycemic agents, which may lead misclassification. Furthermore, we were not able to distinguish between type 1 and type 2 diabetes. Third, we could not clearly ascertain the absence of asymptomatic obstructive coronary artery disease. In addition, the impact of urinary protein and physical activity on our observations could not be assessed, because the information was not uniformly available in our study. Fourth, although we found an independent association between abnormal HOMA-IR and impaired LVGLS, participants with abnormal HOMA-IR group were older and had worse metabolic profiles compared with normal HOMA-IR group, a circumstance that might have affected our observations. Furthermore, we considered a relatively high number of covariates into the multivariable models, which may lead to model overfitting; however, a consistent trend of association between abnormal HOMA-IR and impaired LVGLS was observed in all of the multivariable models, regardless of the covariates considered. Finally, we used internally obtained cutoff value of LVGLS because of the lack of established normal value; therefore, cannot be directly extended to other populations with different demographic composition and risk profiles. However, our cutoff value is comparable to those reported in the previous studies [20, 21].

## Conclusions

The present study demonstrated that insulin resistance was associated with subclinical LV dysfunction, independent of cardiovascular risk factors, LV morphology and diastolic parameters, in normal weight subjects free of overt cardiovascular disease. Our finding may help explain the higher incidence of HF in individuals with insulin resistance.

## Data Availability

The datasets generated and/or analyzed during the current study are not publicly available, but are available from the corresponding author on reasonable request.

## Abbreviations

BMI: Body mass index
DM: Diabetes mellitus
EF: Ejection fraction
HF: Heart failure
HOMA-IR: Homeostatic model assessment of insulin resistance
GLS: Global longitudinal strain
LV: Left ventricle
WC: Waist circumference
